# Incidence and Mechanisms of Coronary Perforations during Rotational Atherectomy in Modern Practice

**DOI:** 10.1155/2020/1894389

**Published:** 2020-11-10

**Authors:** Yen-Hsiang Wang, Wei-Jhong Chen, Yu-Wei Chen, Chih-Hung Lai, Chieh-Shou Su, Wei-Chun Chang, Chi-Yen Wang, Kae-Woei Liang, Tsun-Jui Liu, Wen-Lieng Lee

**Affiliations:** ^1^Ministry of Health and Welfare Taichung Hospital, Taichung, Taiwan; ^2^Division of Interventional Cardiology, Cardiovascular Center, Taichung Veterans General Hospital, Taichung, Taiwan; ^3^Institute of Clinical Medicine, National Yang-Ming University School of Medicine, Taipei, Taiwan; ^4^Department of Life Science, Tunghai University, Taichung, Taiwan

## Abstract

**Objective:**

Heavy calcifications remain formidable challenges to PCI, even for well-experienced operators. However, rotational atherectomy (RA)-induced coronary perforations (CPs) still could not be obviated. This study was to explore incidence and mechanisms of RA-induced CP in real-world practice. Knowing why CPs occur in RA should help operators avert such mishaps.

**Method:**

Patients who received coronary RA from April 2010 to December 2019 with keywords related to perforations were retrieved from database. The procedure details, angiography, and clinical information were reviewed in detail.

**Results:**

A total of 479 RAs were performed with 11 perforations in 10 procedures among 9 patients documented. The incidence of RA-induced CP was 2.1%. The RA vessels were distributed in different territories, including first diagonal branch. Most CPs could be treated conservatively, but prolonged profound shock predisposed to poor outcome. CPs caused by rotawire tip occurred in 18.2% of cases, inappropriately sized burrs in 18.2% of cases, and rotawire damage with subsequent transection and perforation in another 18.2% of cases. A total of 5 (45.5%) perforations were caused by unintended and unnoticed bias cutting into noncalcified plaques (4, 36.4%) or through calcified vessel wall (1, 9.1%). The mechanisms for certain CPs were unique and illustrated in diagrams.

**Conclusion:**

CPs due to RA occur in certain percentage of patients. The mechanisms for CPs are diverse. Wire damage with subsequent transection could occur due to inappropriately repetitive burr stress on the wire body. A significant portion was due to unintended and unnoticed bias cutting into noncalcified plaque or through calcified vessel wall.

## 1. Introduction

Percutaneous coronary intervention (PCI) has become well-recognized modality for treating coronary artery disease, even for complex lesions. However, heavy calcifications remain formidable challenges to PCI, even for well-experienced operators [1]. Heavy calcifications could prevent device delivery, proper preparation of the vessels, stent dislodgement, or underexpansions, causing acute adverse events in the cath lab [2]. The long-term outcomes of stent performance secondary to inadequate calcified lesion preparation were also suboptimal [3]. Heavy calcification could be eccentric or concentric, located at acute angulations or in long lesions with multiple bends, and encountered in acute coronary syndrome. Though intravascular lithotripsy and orbital atherectomy could share part of work to treat coronary calcification [4], rotational atherectomy (RA, Boston Scientific, Natick, MA) has long-lasting history in the coronary interventions and is more familiar to most interventionists. Despite high success and low complication rate, incidences of RA-related coronary perforations (CPs) still could not be obviated in the cath labs [5–7]. Even in certain well-performed procedures, CPs could occur in RA. In high-risk indicated PCI procedures (CHIPs), complex and heavily calcifications are frequently present [8]. CP in these situations usually amounts to severe morbidity or death. Though incidences of CP following RA had been reported before [5, 6, 9], the exact mechanisms for perforation were less well investigated and characterized [6, 10–12], despite several predictors are well known [7]. This study was set out to explore the incidence of CPs and define mechanisms of CPs following RA in a single tertiary care center. Knowing why CPs occur in rotablation should help operators avoid such mishaps in the future.

## 2. Materials and Methods

### 2.1. Study Population

All patients who received RA for coronary lesions from April 2010 to December 2019 with keywords related to perforation, rupture, tamponade, leakage, or pericardial effusion were interrogated from cathroom database and identified by manual inspection. Only CPs caused by RA per se were included but not those by post-RA balloon dilatation or stenting. The procedure details and outcomes of CP were retrieved from the database. The computerized electronic medical chart records of each patient were also reviewed, and relevant clinical information was retrieved and recorded. The study design and protocol were approved by the independent institutional review board (the Institutional Review Board for Human Research and Ethics, Taichung Veterans General Hospital).

### 2.2. Angiographic Characterization and Measurements

Each angiography at the index procedure and, for certain patients, angiography before and after the index treatment were viewed in detail and analyzed on a workstation equipped with software for quantitative analysis of angiograms (Rubo DICOM Viewer, version 2.0, build 170828, Rubo Medical Imaging, Aerdenhout, the Netherlands). The SYNTAX scores before and after PCI were calculated using the standard calculator software available at the official website. As a tradition in our cath lab, both cine and in-procedure fluoroscopy were stored in our PACKS system. All the images, especially those surrounding RA and CP, were scrutinized for subtle wire and burr movements to identify causes of CPs. The types of CP were categorized by the modified Ellis classification system [13]. The numbers of burr run before lesion crossing and those for downstream plaques after crossing (thus, the perforation site) were counted from all the stored fluoroimages and recorded.

All PCIs were performed by certified interventional cardiologists in accordance with the standard practice at our cath lab. Patients were pretreated with a standard dose of aspirin and clopidogrel (or ticagrelor). Calcium channel blocker and nitrate were also used to prevent coronary artery spasm. Heparin was administered to maintain an activated clotting time (ACT) of ≥300 seconds during procedure. The decision to do RA was guided by standard practice but also at the discretion of the operator. The indications for RA could be primary (for heavy and circular/rotating intimal calcification) or secondary as bail-out procedure (for device undilatable, using 1 : 1 balloon up to 16 bars, or uncrossable lesions). Prior to RA, a 0.009-inch floppy or extra-support RotaWire™ with tip gently J-curved by the operator was advanced through the lesion using the wire-exchange technique through a microcatheter. A bolus of 1,200–1,600 ug of isosorbide dinitrate was given intracoronarily prior to the start of RA, during which time normal saline mixed with heparin and isosorbide dinitrate was slowly infused. RA was implemented using the Rotablator™ RA system (RotaLink Plus), starting with a 1.25 (for most bail-out indications and vessels with very small lumen) or 1.5 mm burr at a speed of 180,000–200,000 rpm, and this was often supplemented by another burr one-size bigger. Each burr advance time was less than 20–30 seconds. The times of polishing after burr passing varied and depended on the operator's choice. In case of dissections by ballooning attempts but vessel remained undilatable, RA could proceed if this treatment was considered technically feasible. For patients who needed side branch (SB) rotablation, the sequence of RA of SB or main vessel (MV) was determined by which vessel was more critically diseased and potentially jeopardized if not treated first, and also at the discretion of the operator. After the vessel and blood flow in the first treated vessel were secured, rotawiring of another vessel was made and followed by RA of the second vessel. After accomplishment of RA, workhorse wire replaced RotaWire^™^ using the same wire-exchange technique through the microcatheter and the procedure proceeded with balloon angioplasty with or without stent implantation. Completion of RA was defined as full debulking of the target lesion without premature termination of RA before proceeding to subsequent treatment. After stent implantation, dual-antiplatelet therapy with aspirin (100 mg/day) and clopidogrel (75 mg/day; or ticagrelor 90 mg twice day) was continued for at least 12 months after DES implantation. Use of IVUS for vessel sizing was at the discretion of the operators and not mandatory. In case of complications during the procedure, the diagnosis and emergent managements depended on the specific complication and performed according to standardized protocols. Burr entrapment was treated firstly with attempts of forceful burr extraction and then, if not successful, by 5-in-6 daughter-in-mother catheter technique. The heart team was activated once a major complication occurred and did not respond to conventional measures. Cardiogenic shock was defined as systolic blood pressure lower than 90 mm Hg after appropriate fluid supplement together with clinical or laboratory evidence of hypoperfusion. Patients who remained in the similar or worse status despite high dose vasopressor supporting greater than 0.5 *μ*g/kg/min of norepinephrine or equivalent were referred to as having “profound /refractory shock..”

### 2.3. Clinical Outcomes

The cardiovascular major adverse cardiac events (CV MACEs) were defined as cardiovascular death, nonfatal myocardial infarction, target lesion or target vessel revascularization, and followed up till the first month after index procedure.

### 2.4. Statistics

Data of continuous variables are expressed as mean and distribution ranges. The categorical variables are presented as number and frequency. The statistical analyses were performed using the IBM SPSS statistical packages software for Windows, version 24.0.0.0 (IBM corp., New York, US).

## 3. Results

During the study period, a total 479 RAs were performed at our cath labs and a total of 11 perforations in 10 procedures among 9 patients secondary to RA per se were documented with an incidence of 2.1% (10/479). The mean age was 75.9 (66–85) years. The demographic data of these 9 patients are presented in Supplementary Table 1.

One patient (Case 7) suffered from 2 perforations (LCX proper and far distal LCX) in 1 procedure, whereas another 1 patient (Case 8) suffered from 2 perforations in 2 separate procedures as she had to endure bail-out procedure for another vessel after the first one precipitated shock due to RA-related CP in one vessel. The procedure details are presented in Supplementary Table 2.

**Figure 1:**
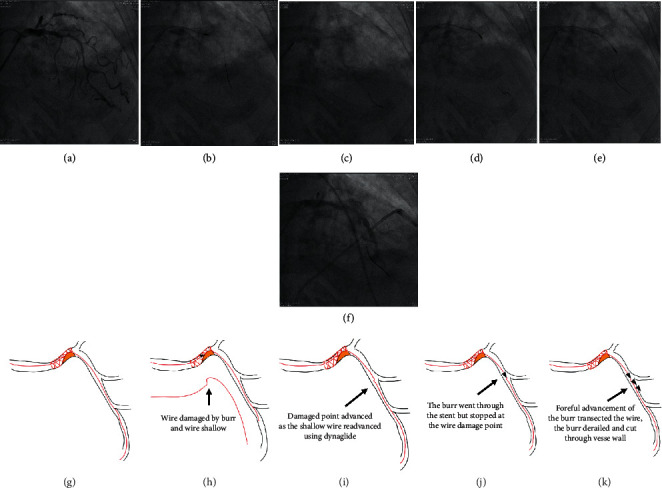
Illustration for vessel perforation in Case 4.

**Figure 2:**
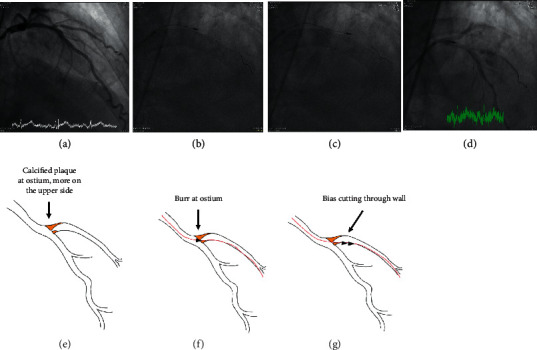
Illustration for vessel perforation in Case 3.

**Figure 3:**
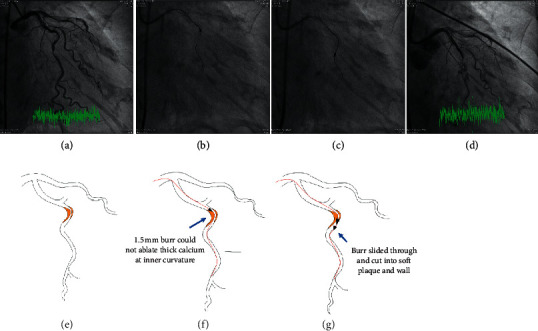
Illustration for vessel perforation in Case 8, procedure for LCX.

**Figure 4:**
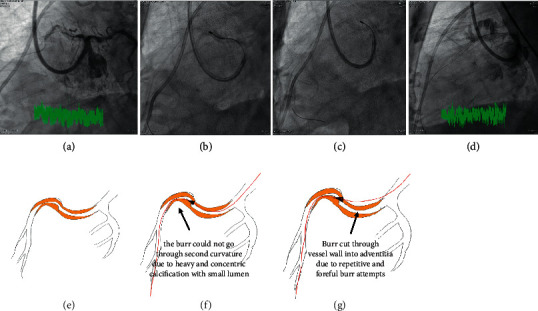
Illustration for vessel perforation in Case 8, procedure for LAD.

The indication for RA, exact location, Ellis type, mechanisms, device details, and consequences of the 11 CPs are presented in Table 1. CP caused by rotawire tip trauma occurred in 2 (18.2%), inappropriately sized burrs in two (18.2%), and wire damage followed by wire transection, derailment of burrs, and perforation in another 2 (18.2%) cases. The mechanism for wire damage and subsequent transection during RA for underexpanded stent in Case 4 was very unique and is illustrated in Figure 1. A total of 5 (45.5%) perforations were caused by unintended and unnoticed bias cutting into noncalcified plaques (4, 36.4%) or through calcified wall (1, 9.1%). The mechanisms for unintended bias cutting and subsequent perforations presented differently in different individuals, and those for Case 3, Case 8 first procedure, and Case 8 second procedure are illustrated in Figures 2–4, respectively. In CP secondary to bias cutting, extra burr runs on downstream plaques after lesion crossing were clearly noticed and responsible for the mishaps. In Case 8 second procedure, the numbers of burr run responsible for bias cutting through the wall calcification were enormous, implying that the downstream lesion was unusually hard. As a consequence of perforation, Ellis types 1, 2, or 5 perforations occurred in 7 out of 11 procedures (63.6%) and type 3 in 4 (36.4%). Hypotension occurred in 2 (20.0%), and profound shock in another 3 procedures (30.0%).

The management of acute perforation and treatment outcomes of these 10 procedures is shown in Tables 2 and 3. Prolonged balloon occlusion was used in 2 (20.0%), coil embolism in 3 (30.0%), and stent grafting in 5 (50.0%). The perforation was successfully sealed in 9 but 1 (10.0%), who was sent for emergent open-heart surgery for bypass and repair in stable condition (Case 4). Another patient underwent elective CABG for occluded vessel (Case 2) after the perforation successfully sealed by coil embolism. Pericardiocentesis was needed in 5 procedures (50%) and bail-out IABP in 2 (20.0%, the same patient, 2 separate occasions). Rotablation for the treated vessel could be completed in 6 (60.0%) of these procedures.

One patient (Case 8, Table 3) died in the hospital due to prolonged profound shock following vessel perforations in 2 separate vessels on 2 procedures despite the large perforations could be successfully sealed after prolonged endeavor. The others could be discharged from hospital with stable vital signs and mean hospital days of 13.8 (2–54), including the one undergone emergent open heart. The 30-day MACE was marked by an acute event. One patient (Case 7) was readmitted to hospital 4 days later due to acute stent thrombosis in the LCX with cardiogenic shock. He was treated with emergent revascularization and IABP implantation, and discharged from hospital in stable condition 9 days after admission.

## 4. Discussion

In summary, our study found that (1) the incidence of CP secondary to RA per se was 2.1% at our center; (2) a large proportion of CPs could be treated conservatively except for those with resultant frank perforation and prolonged cardiac shock; and (3) after reviewing the session cine and stored fluoroimages in detail, four mechanisms of CPs during RA were revealed and consisting of rotawire tip trauma, using inappropriately sized burrs, rotawire damage with subsequent transection, and unintended bias cutting into noncalcified or through calcified vessel walls at acute turns. Unintended bias cutting accounted for a significant proportion of burr-induced CPs and happened without being noticed in procedure while the operators chased for more debulking or procedure success.

CP is one of the nightmares during PCI, associated with worse acute and intermediate term results, and occurs at an incidence of 0.17–0.68 [9, 14–16]. The risk factors for CPs in general PCI include old age, female, high SYNTAX scores, DM, renal failure, CTO PCI, and using RA. The incidence of CPs during RA varied in the literature and reported to be 0.4–4.0% [5–7, 17–19]. CP during RA was associated with even worse short- and long-term results [14, 20, 21]. Nowadays, more and more RA was done for CHIP and off labeling, [8] which was associated with more adverse events in comparison with on-label use [17]. However, these CHIP patients are also highly vulnerable to complications during procedure. Therefore, how to properly evaluate the patient and lesion characteristics and how to obviate the caveats to RA-induced CP are of paramount importance for uneventful RA and PCI. In this study, we found an RA-induced CP incidence of 2.1% in our cath lab, which was in the average of reported series. We do believe these figures depend on how the operators select patients and treat lesions. In our cathroom, every single independent operator is capable of doing RA, whereas some operators were more experienced and cautious than others. On the other hand, more experienced operators were more willing to challenge difficult lesions based on their judgement. This could be reflected by the fact that more RA-induced CP occurred in recent years and carried out by very-experienced interventionists.

Despite the fact that CP could occur in RA, full mechanisms have not been precisely characterized before [6, 10–12, 22]. Our study population might be large enough to warrant an in-depth analysis. As a tradition in our cath lab, both cine and in-procedure fluoroscopy were stored in our PACKS system and our cathroom was equipped with X-ray machines generating good image qualities. These two advantages enable us to track subtle images during procedure post hoc and to fully reveal the causes of RA-induced CP. A total of four mechanisms were identified, including rotawire tip injury, using inappropriately sized burr, wire damage with subsequent transection, and unintended and unnoticed bias cutting into noncalcified plaque or through calcified vessel wall. Though these mechanisms were not strange to most operators, certain cases were generated in very particular occasions, by unusual device manipulations and deserve particular attentions.

Inappropriately sized burr consists of two different scenarios: using even the smallest 1.25 mm burr may still be too big for a relatively small-sized vessel, and starting with a bigger burr for certain complex lesion due to economic reason may not be a good choice. As we are moving toward more complex and calcified lesions, device-uncrossable and undilatable lesions became more frequently encountered and the operators were enticed to do RA. However, some of these vessels are relatively small, and even the smallest 1.25 mm might be relatively large for the vessel and cause vessel perforations accordingly. On the other hand, starting with a 1.5 mm burr was inappropriate for the very thick calcium layer at the inner curvature of the acute turn in Case 9. Failure to ablate this calcium let the burr slide through the acute curvature and made bias cutting into noncalcified vessel wall and hence large perforation beyond the acute turn.

In terms of wire damage and subsequent transection, passing burr through acute angulations more than 90°, like those at the LM-LCX junction, to treat distal lesions, should be well planned beforehand and carried out cautiously. Floppy rotawires are usually not strong enough to track the burr, and forceful advancement of burr through acute angulation might transect the wire. Using an extra-support rotawire should be considered in this circumstance. However, wire bias and cutting bias of the burr into the inner curvature of the acute angulation should be carefully weighed to prevent too much bias cutting and perforation. This mechanism accounted for one perforation in our population (Case 5, at LCX proper) and could be prevented if the operator had good planning ad hoc. In our population, another kind of wire damage and transection occurred during RA to treat underexpanded stent (Case 4, mechanism illustrated in Figure 1). This mechanism for perforation is very unique and should be born in mind whenever burr could not travel along the rotawire at a point when it should go without resistance in difficult cases, in which wire damage could have happened after prolonged and repetitive burr attempts on the same point of the wire. Rotawire fracture was also reported to occur in during burr removal [23].

The most important findings of our study should be identifications of unintended and unnoticed bias cutting as an important cause for vessel injury and perforation in a nonnegligible proportion of patients (45.5%). Though wire bias was previously considered as cause of vessel perforation, we do believe that bias cutting is the fundamental mechanism as wire bias is omnipresent except in straight vessels. Intended wire bias and bias cutting are sometimes used purposefully to treat heavy eccentric calcification on the inner curvature of vessel bend. However, unintended bias cutting is responsible for nonpurposeful injury to noncalcified plaque and perforations, which could be averted if noticed before bias cutting goes too far. The causes of vessel perforation in these cases mostly went unnoticed in procedure while the operators chased for more plaque debulking after lesion crossing or made too much attempts before lesion crossing. Only after detailed and in-depth review of the cine and stored fluoroscopy as we did here could the mechanisms fully unveil. In our cases with CP secondary to bias cutting, extra burr runs on downstream plaques after lesion crossing were clearly noticed. In Case 8 second procedure, the numbers of burr run responsible for bias cutting through the wall calcification were enormous, implying that the downstream lesion was unusually hard and the operators were determined to succeed. Perforations secondary to this mechanism mostly were not caused by poor technique, but lack of knowledge that how bias cutting could occur. Three of most educative cases were illustrated in diagrams in our study to help full understanding. Bias cutting is caused primarily by vessel bending and exaggerated by nonequal distribution of calcium around the wall and ex-support rotawire. The hints to bias cutting into noncalcified vessel wall and potential complication were deviation of burr and wire from existing calcium in the vessel and traveling of burr out of vessel trajectory. The trick to minimize CPs in these is to limit burr pass and polishing times to avoid too much tissue damage and/or closely watching the relationship between the rota burr and the calcium in the vessel wall and stopping further burr attempts once the burr deviates from the calcium or out of vessel route trajectory. By adopting these tricks, we in our cath lab have done several other complex lesions with acute bends without complications in recent days, which were not possible before. To our knowledge, this current study should be the first to fully analyze and illustrate diagrammatically the mechanisms for RA-related CP which were not well appreciated before.

Study limitations. This was a single-center study with complications ascribed to individual operators with different rotablation experiences. Some perforation cases might be viewed as preventable by rotablation experts or contradictory to the procedure in post hoc review. However, our study patient cohort and cases did reflect the real-world practice for patients with angiographic heterogeneity and by various operators with their own clinical judgement. In country where bypass surgery was less likely to be accepted by patients such as in ours, operators, even the most experienced ones, were more likely to challenge very complex lesions and encounter formidable complications. Therefore, these complications convey important educative values. Despite this was a single-center study, the patient cohort was large enough and RA-related CP of different mechanisms could be identified after an in-depth analysis. These are the reasons why we conducted this study to let all operators to comprehend the knowledge and skills of RA in complex lesions in order not to repeat such mishaps in the future.

## 5. Conclusion

CPs due to RA occur in certain percentage and may vary according to patient selection and lesion complexity to treat. Four mechanisms, consisting of rotawire tip injury, using inappropriately sized burr, rotawire damage with subsequent transection, and unintended bias cutting into noncalcified plaque or through calcified vessel wall, were identified. Wire damage with subsequent transection could occur due to inappropriately repetitive burr stress on the wire body. Most of the last mechanism goes unnoticed during procedure. Limiting the numbers of burr pass and/or closely watching the relationship between the rota burr and the calcium in the vessel wall, and stopping further burr attempts once the burr deviates from the calcium or out of vessel trajectory could help avert such burr-induced CPs.

(Legend for Figure 1) A drug-eluting stent was placed in calcified mid-LAD but underexpanded at distal end, accentuating the acute bend (Panels A and G). Due to very-hard consistency, insufficient floppy rotawire support, and acute bend, the wire was damaged and an acute bend was created at the repetitive stress point by burr (Panels B and H). As the rotawire became too shallow after repetitive burring, it was readvanced under the vessel using dynaglide, along with the wire damage point (Panels C and I). After more attempts and burring along the nondamaged part of the wire, the burr successfully went through the underexpanded stent but stopped at the wire damage point (Panels *D* and J). The operator did not know the mechanism and further forceful attempts transected the wire, derailed the burr (Panels *E* and K), and perforated the vessel (Panel F).

(Legend for Figure 2) The tight and heavily calcified ostial lesion at first diagonal branch was not device-crossable. However, the calcium soon became eccentric beyond ostium (Panels A and E). The operator put in a floppy rotawire. The burr passed through the ostium without too many difficulties (Panels B and F). However, the burr soon bias cut into the noncalcified lower vessel wall beyond the ostium (Panels C and G) and perforated the vessel after extra runs (Panel D).

(Legend for Figure 3) The 85-year-old female had triple vessel disease but was too old and morbid for bypass surgery. The operator decided to treat LCX with RA first. There was heavy concentric calcification, thicker at inner side of the curvature, at first acute turn (Panels A and E). An extra-support rotawire was put in, intended to bias cut the calcium at inner side. However, the procedure was difficult and made the wire shallow after repetitive attempts. Furthermore, the starting 1.5 mm burr was too big and failed to ablate the thick calcium at inner side of the curvature (Panels B and F), but it slid over the thick calcium and bias cut into the noncalcified vessel wall beyond the curvature (Panels C and G), and it produced large vessel perforation and cardiogenic shock after 8 more runs (Panel D).

(Legend for Figure 4) Due to persistent hypotension and dependence on IABP, bail-out PCI for LAD was intended to save the patient. The concentric heavy calcification in proximal-to-middle LAD was S-shaped with very small lumen (Panels A and E). As the burr could not go through the heavy calcium at the second curvature, the wire was exchanged to an extra-support rotawire (Panels B and F). Despite these, the 1.25 mm burr still failed to ablate heavy calcium at the second curvature but made bias cutting through the heavily calcium in the upper wall of first curvature and finally cut into adventitia after enormous numbers of burr attempts (Panels C and G). The large perforation and profound shock irreversibly worsened the existing hypotension.

## Figures and Tables

**Table 1:** Indication for RA and perforation location, type, mechanisms, and consequences.

Case	Perforation location	Perforation	RA indication, mechanism of perforation, type of rotawire, numbers of burr run	Shock
1	LAD-apical branch	Type 5	Primary indication; vessel trauma by floppy rotawire tip	-
2	RCA-M-D junction, acute turn with small radius	Type 2	Bail-out indication; bias cutting into noncalcified side beyond acute turn, burr deviated from calcium (1.25 mm burr, burr to artery ratio 0.43, floppy rotawire); numbers of burr run = 19 before crossing and another 13 after crossing caused perforation	-
3	First diagonal, body	Type 2	Bail-out indication; bias cutting into noncalcified side beyond D1 ostium, burr deviated from calcium (1.25 mm burr, burr to artery ratio 0.48, floppy rotawire); numbers of burr run = successful crossing in 1 and another 4 after crossing caused the perforation	Hypotensive
4	LAD-M, underexpanded stent edge	Type 3	Bail-out indication; rotawire damage by burr, wire transection and burr derailment (floppy rotawire, 1.25 mm burr); numbers of burr run = 11, all pushed forcefully against the lesion for few seconds (thus damaged the wire)	Profound shock, short-duration
5	LCX-PMJ, 90-degree acute turn	Type 3	Primary indication; wire too shallow, rotawire damage and transection by burr, burr derailment (extra-support rotawire, 1.25 mm burr); numbers of burr run = 3	No, limited by previous CABG
6	First diagonal, body	Type 2	Bail-out indication; bias cutting into noncalcified side beyond D1 ostium, burr deviated from calcium (1.25 mm burr, burr to artery ratio 0.54, floppy rotawire); numbers of burr run = successful crossing in 1 and another 4 after crossing caused the perforation	-
7	LCX-far distal	Type 5	Vessel trauma by floppy rotawire tip due to no release of brake during dynaglide,	-
	LCX-distal	Type 2	Bail-out indication; smallest 1.25 mm rota burr too large for small-sized mid-LCX (burr to artery ratio 0.82, floppy rotawire); numbers of burr run = 5 before lesion crossing and another 11 after crossing caused the perforation	-
8	LCX-M, acute bends	Type 3	Primary indication; 1. Start with too big (1.5 mm) burr, could not ablate calcium at inner curvature of first acute turn (burr to artery ratio 0.64, extra-support rotawire) 2. Bias cutting into noncalcified side beyond first turn, burr deviated from calcium; numbers of burr run = 22 before crossing and another 8 after crossing caused the perforation	Profound shock, long duration
8	LAD-P, LAD-MDJ, S-shaped bends	Type 3	Primary indication; bias cutting through calcium into adventitia of proximal curvature (LAD-P) as burr could not go down the very-hard second curvature, burr deviated from calcium (1.25 mm burr, burr to artery ratio 0.45, floppy followed by extra-support rotawires); numbers of burr run = 32 for the floppy and 55 for the extra-support rotawire	Further shock, long duration
9	RCA-P-M junction with acute turn	Type 2	Primary indication; bias cutting into noncalcified inner curvature side of the acute turn (1.5 and 1.75 mm burrs, burr to artery ratio 0.52, floppy rotawire), numbers of burr run = 18 for the 1.5 mm burr and another 11 for the 1.75 mm burr across the perforation site	Hypotensive

**Table 2:** Management of acute perforation and treatment outcomes.

Case	Management of perforations	IABP/ECMO	Pericardiocentesis	Rota completion	Stenting	Management outcome	CABG	In-hospital mortality	In-hospital complication
1	Coil embolism	−	−	+	+	Successfully sealed	−	−	−
2	Coil embolism	−	−	−	−	Successfully sealed	Elective	−	Hemothorax
3	Stent graft	−	+	+	+	Successfully sealed	−	−	−
4	BC occlusion	−	+	−	−	Failed to seal, unable to deliver stent graft	Emergent	−	Acute cholecystitis
5	Stent graft	−	−	−	+	Successfully sealed	−	−	Lower GI bleed
6	BC occlusion	−	−	+	−	Successfully sealed	−	−	−
7	Coil embolism	−	−	+	+	Successfully sealed	−	−	−
8	Stent graft	IABP	+	+	+	Successfully sealed, prolonged profound shock, intubation	−		Shock
8	Stent graft	IABP	+	−	+	Successfully sealed, further prolonged shock	−	+	Profound shock
9	Stent graft	−	+	+	+	Successfully sealed	−	−	−

**Table 3:** Procedure events and clinical outcomes (10 procedures in 9 patients).

Hypotension (*N*, %) (*N* = 10)	2 (20.0%)
Profound shock (*N*, %)	3 (30.0%)
Emergent IABP (*N*, %)	2 (20.0%)
Emergent pericardiocentesis (*N*, %)	5 (50.0%)
Emergent management (*N*, %) (*N* = 10)	
Balloon occlusion	2 (20.0%)
Coil embolism	3 (30.0%)
Stent grafting	5 (50.0%)
Failure to seal perforation (*N*, %)	1 (10.0%)
CABG (*N*, %) (*N* = 9)	
Emergent	1 (11.1%)
Elective	1 (11.1%)
Die on table (*N*, %)	0
Acute CIN (*N*, %)	2 (22.2%)
In-hospital CV MACE (*N*, %) (*N* = 9)	6 (66.7%)
In-hospital CV death	1 (11.1%)
In-hospital MI	6 (66.7%)
In-hospital stent thrombosis	0
In-hospital stroke	0
In-hospital TLR	0
In-hospital TVR	1 (11.1%)
Total hospital days	13.8 (2–54)
30-day CV MACE (*N*, %) (*N* = 8)	1 (12.5%)
30-day death	0
30-day CV death	0
30-day nonfatal MI	1 (12.5%)
30-day stent thrombosis	1 (12.5%)
30-day stroke	0
30-day TLR	1 (12.5%)
30-day TVR	0

## Data Availability

The data used to support the findings of this study are available from the corresponding author.

## Abbreviations

CIN: Contrast-induced nephropathy
MACE: Major adverse cardiovascular event
TLR: Target lesion revascularization
TVR: Target vessel revascularization.

## Supplementary Materials

Supplementary Materials Supplementary Table 1: demographic data of all patients with coronary perforations during rotational atherectomy. Supplementary Table 2: angiographic and procedural details during rotational atherectomy.
