# Supplementary material for: Incidence and Mechanisms of Coronary Perforations during Rotational Atherectomy in Modern Practice
Source: J Interv Cardiol. 2020 Nov 10;2020:1894389. doi: 10.1155/2020/1894389 (PMC7673942; doi:10.1155/2020/1894389)
Supplement: Supplementary Materials — Supplementary Table 1: demographic data of all patients with coronary perforations during rotational atherectomy. Supplementary Table 2: angiographic and procedural details during rotational atherectomy. [file 1894389.f1.zip › 1894389.f1/supplementary Table 1 (1).docx]

Supplementary Table 1. Demographic data of all patients with coronary perforations during rotational atherectomy

Sex (M/F) 4//5

Age (years) 75.9 (66-85)

Clinical Diagnosis (N, %) 9

stable angina 3 (33.3 %)

unstable angina 1 (11.1 %)

NSTEMI 5 (55.6 %)

STEMI 0

ischemic cardiomyopathy 0

Cardiogenic shock 0

Hypertension (N, %) 7 (77.8 %)

Diabetes (N, %) 5 (55.6 %)

Hypercholesterolemia 4 (44.4 %)

Chronic renal disease 2 (22.2 %)

End-stage renal disease 2 (22.2 %)

Previous stroke 4 (44.4 %)

Peripheral arterial diseases (N, %) 0

Previous PCI 4 (44.4 %)

Previous CABG 1 (11.1 %)

Baseline LVEF (%) 50 (36-55)

Lab data

Hemoglobin (g/dl) 13.4 (8.8- 13.4)

BUN (mg/dl) 22 (13-32)^*^

Cr (mg/dl) 1.1 (0.5-1.7)^*^

Cholesterol (mg/dl) 148.1 (71-215)

HDL-chol (mg/dl) 46.9 (28-100)

LDL-chol (mg/dl) 81.4 (19-140)

FBS (mg/dl) 185 (85- 616)

HbA1c (mg/dl) 7.3 (5.4-14.2)

Total CK (U/L) 105 (40-154)

CK-MB (U/L) 8 (5-11)

Troponin (ng/ml) 33.7 (0.03-171.2)

CAD vessels (N, %)

Single-vessel disease 3 (33.3 %)

Double-vessel disease 4 (44.4 %)

Triple-vessel disease 2 (22.2 %)

Left anterior descending 7 (77.8 %)

Left circumflex 4 (44.4%)

Right coronary artery 6 (66.7 %)

Plus left main 0

^*^patients not in ESRD;

Abbreviations: NSTEMI: non-ST-elevation myocardial infarction; STEMI: ST-elevation myocardial infarction; PCI, percutaneous coronary intervention; CABG, coronary artery bypass graft; LVEF, left ventricular ejection fraction
