# Supplementary material for: Incidence and Mechanisms of Coronary Perforations during Rotational Atherectomy in Modern Practice
Source: J Interv Cardiol. 2020 Nov 10;2020:1894389. doi: 10.1155/2020/1894389 (PMC7673942; doi:10.1155/2020/1894389)
Supplement: Supplementary Materials — Supplementary Table 1: demographic data of all patients with coronary perforations during rotational atherectomy. Supplementary Table 2: angiographic and procedural details during rotational atherectomy. [file 1894389.f1.zip › 1894389.f1/supplementary Table 2 (1).docx]

Supplementary Table 2. Angiographic and procedural details during rotational atherectomy

Rotablation vessels (N, %) 10

LAD proper 3 (30.0 %)

First diagonal 2 (20.0 %)

LCX proper 3 (30.0 %)

RCA proper 2 (20.0 %)

Access site (N) 10

Radial (N, %) 6 (60.0 %)

Femoral (N, %) 4 (40.0 %)

Guide size (N) 10

6F (N, %) 4 (40.0 %)

7F (N, %) 6 (60.0 %)

Syntax score 26 (11-42)

Syntax score post-PCI 18 (0-42)

Syntax score gain 8 (0-25)

Rotablation vessel characteristics

Total lesion length (mm) 35.1 (4.1-80.4)

Reference diameter (mm) 2.7 (2.0 - 3.5)

Minimal lumen diameter (mm) 0.6 (0.0- 1.5)

Heavy calcification (N, %) 10 (100%)

Indication for rotablation (N, %) 10

Primary 5 (50.0 %)

Bail-out 5 (50.0 %)

Tortuosity/ acute bend (N, %) 7 (70.0 %)

Ostial lesion (N, %) 3 (30.0 %)

Bifurcation (N, %) 4 (40.0 %)

Chronic total occlusion 1 (10.0 %)

Under-expanded stent 1 (10.0 %)

ACC/AHA lesion (N, %) 10

B2 4 (40.0 %)

C 6 (60.0 %)

Rotablation completed (N, %) 6 (60.0 %)

Largest burr size (N, %)

1.25mm 6 (60.0 %)

1.5mm 3 (30.0 %)

1.75mm 1 (10.0 %)

Rota wire type (N, %)

Floppy 7 (70.0 %)

Extra-support 3 (30.0 %)

Lesion stenting 7 (70.0 %)

Total procedure time (min) 176.4 (120.0- 342.0)

Total fluoro time (min) 52.1 (22.0- 92.5)

Total contrast dose (min) 196 (90-300)

Abbreviations: LM, left main coronary artery; LAD, left anterior descending artery; LCX, left circumflex artery; RCA, right coronary artery; BMS, bare metal stent; DES, drug-eluting stent
